# Are Market GM Plants an Unrecognized Platform for Bioterrorism and Biocrime?

**DOI:** 10.3389/fbioe.2019.00121

**Published:** 2019-05-29

**Authors:** Siguna Mueller

**Affiliations:** Independent Researcher, Kaernten, Austria

**Keywords:** unrecognized bio-weapons, plants as attack vectors, GMO authentication, unauthorized GMOs, GMO counterfeiting, clandestine manipulation of biological mediums, covert manipulation of non-GMOs

## Abstract

This article discusses a previously unrecognized avenue for bioterrorism and biocrime. It is suggested that new gene editing technologies may have the potential to create plants that are genetically modified in harmful ways, either in terms of their effect on the plant itself or in terms of harming those who would consume foods produced by that plant. While several risk scenarios involving GMOs—such as antibiotic resistant pathogens, synthetic biology, or mixing of non-GMO seeds with GMO seeds—have previously have been recognized, the new vulnerability is rooted in a different paradigm—that of clandestinely manipulating GMOs to create damage. The ability to actively inflict diseases on plants would pose serious health hazards to both humans and animals, have detrimental consequences to the economy, and directly threaten the food supply. As this is the first study of this kind, the full scope and impact of suck attacks—especially those involving the intended misuse of technologies such as gene-drives—merits further investigation. Herein, the plausibility of some of the new risks will be analyzed by, (1) Highlighting ownership and origination issues (esp. of event-specific GM-plants) as unrecognized risk factors; (2) Investigating the unique role of GMOs, why—and how—certified GMOs could become a new venue for such attacks; (3) Analyzing possible dual-use potentials of modern technologies and research oriented toward the advancement of GMOs, plant breeding and crop improvement. The identification and analysis of harmful genetic manipulations to utilize (covertly modified) plants (GMOs and non-GMOs) as an attack vector show that these concerns need to be taken seriously, raising the prospect not only of direct harm, but of the more likely effects in generating public concern, reputational harm of agricultural biotechnology companies, law-suits, and increased import bans of certain plants or their derived products.

## 1. Motivation

According to their current definition, biological weapons achieve their intended target effects through the infectivity of disease-causing infectious agents. The CDC^1^ defines bioterrorism as the deliberate release of viruses, bacteria, or other agents used to cause illness or death in people, and also in animals or plants (Jansen et al., 2014).

While there has been much focus on traditional infectious agents such as bacteria and viruses, this article describes a previously underappreciated vulnerability. It is suggested that novel gene editors and other scientific advances allow for a clandestine manipulation of GM plants^2^ already on the market. These concerns are different than those previously raised about GMOs (which don't fit the category of “intended targeted effects”).

Whether it is consternation about weediness, the development of super-bugs and antibiotic resistance, influences on human health, or concerns about unavoidable and irreversible impacts on “neighboring farmers, regions, and countries” (Brown, 2017) through technologies with scaled-up capacities such as gene drives, previous concerns about GMOs centered on the understanding that the malignant effect would occur as an unintended consequence. In contrast, this article considers modern gene editing technologies as possible platform for a hostile manipulation of GMOs.

In the context of emerging agricultural technologies via infectious genetically modified viruses engineered to edit crop chromosomes directly in the field, Reeves et al. (2018) describe a “relatively benign hypothetical targeted weaponization scenario” via “horizontal environmental genetic alteration agents.” In this example, the “targeted weaponization” relies on the fact that “the released virus-infected insects may survive longer” than stipulated. The effect would be that “fields experience a food and seed shortage.” The concern of these authors is about more serious forms of attack.

The most skillful attacks don't rely on natural circumstances and happenstance. As detailed by Berns et al. (2012), “We are in the midst of a revolutionary period in the life sciences. Technological capabilities have dramatically expanded, we have a much improved understanding of the complex biology of selected microorganisms, and we have a much improved ability to manipulate microbial genomes. …However, there is also a growing risk that the same science will be deliberately misused and that the consequences could be catastrophic.”

This article describes a previously unrecognized form of biocrime—the weaponization of GMOs. This may be best explained by the following—already familiar—predicament of seed contamination which once again made the news. On February 7, 2019, an unauthorized GMO strain was identified in Europe, mixed in with the natural seed bought from Bayer-Monsanto. By the time an official recall was issued, some of the seeds had already been planted, covering 8, 000 ha in France and 3, 000 ha in Germany.

“Unauthorized GMOs” (UGMOs) are GMOs that are released in the market of a certain jurisdiction without prior authorization (Arulandhu et al., 2016). This may include those approved in some countries but not in others (e.g., due to some threshold restrictions), but also those that have not (yet) received any regulatory approval in any country (e.g., because they are still in the process of laboratory and field trials).

Instead of accidental and unintended mingling of merely unauthorized GMOs, it is suggested that existing GMOs may be exchanged in clandestine, with the intent to create damage. Thus, instead of attacks on plants, the possibility is considered that malignant genetic manipulations may turn plants themselves into harmful attack vectors. This possibility opens up an unrecognized avenue for bioterrorism or biocrime—either by maliciously modifying a natural plant or (perhaps more perniciously) sabotaging a previously approved GMO. Compounding the problem is that such an introduction will be difficult to detect. This is particularly the case when nobody is looking for such manipulations. However, an additional factor is just as important. It will be shown that current GMO identification and authentication techniques suffer from critical vulnerabilities that may be misused. Additionally, several attack scenarios are analyzed, how perpetrators may attempt to introduce harm, either on the plant itself or in terms of those who would consume foods produced by the plant. Some of these need to be taken seriously, also because of their impact on public perception and acceptability of GMOs and agricultural biotechnology companies.

**Figure 1:**
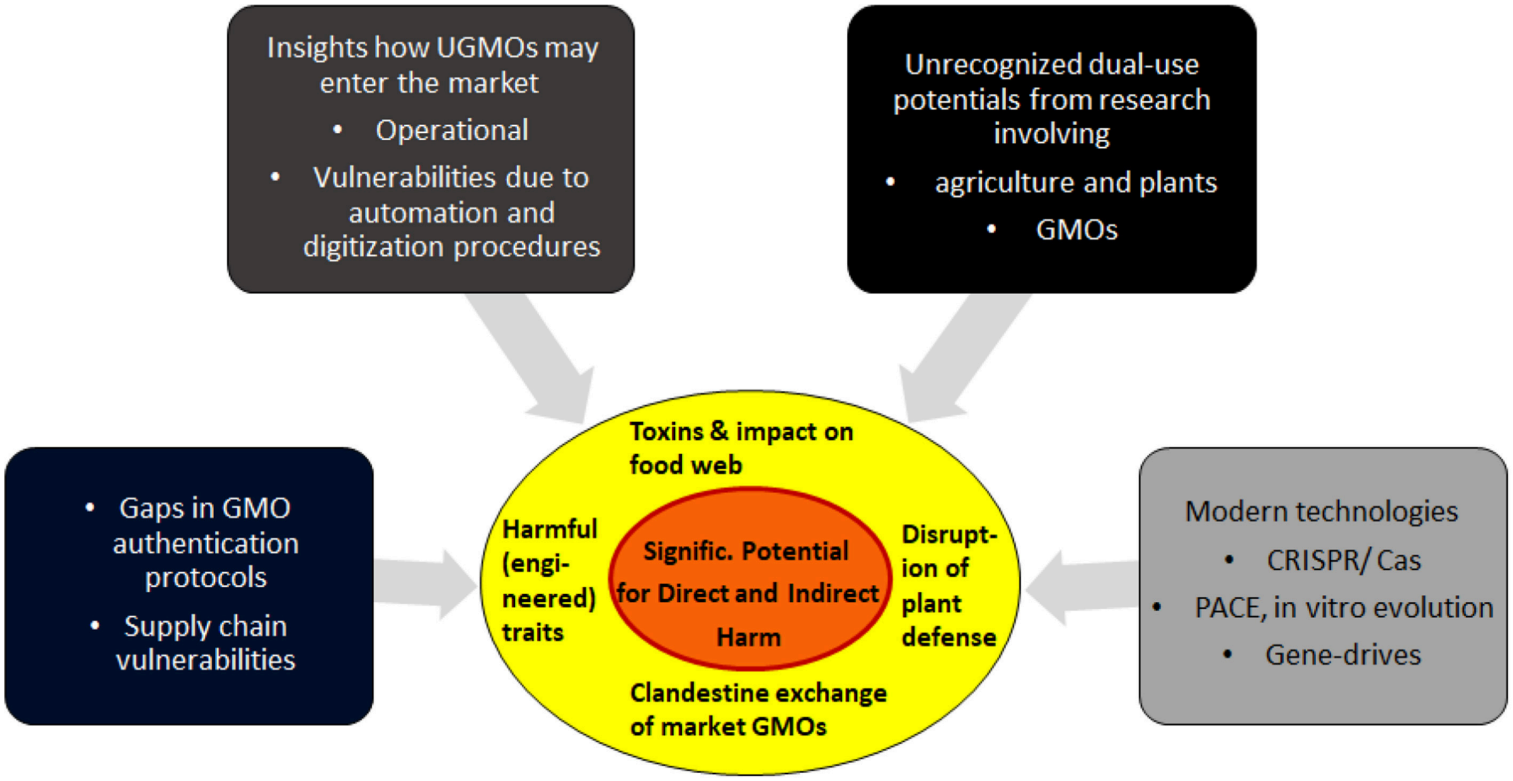
Biocrime in form of clandestine manipulations of GMOs: A summary of the main contributing factors. Details and feasibility of these vulnerabilities are analyzed in the following sections.

*Overview* section 2 describes the most critical elements of the new vulnerabilities. Section 3 focuses on dual-risk potentials arising from research on GMOs and plants, and describes specific aims how attackers may try to exploit GMOs as weapons. Section 4 finishes with discussions on practical aspects, the feasibility of such attacks, and some open questions. For a summary of the interplay of the various factors, see Figure 1.

## 2. Examining the Core vulnerabilities

### 2.1. The Problem of Detecting UGMOs May Become an Entry Point for the Distribution of GMO Weapons

Although GMOs continue to be rigorously investigated and are regulated by The International Regulations and the Codex guidelines (see e.g., FAO/WHO, 2003; Johnson, 2014), the realization of unified, international regulations has proven challenging. Bar-Yam et al. (2014) describe the situation in the US alone as, “a regulatory system marked by fragmentation, lack of coordination and different standards for different types of products.” Even worse, in 2013 for less than 30% of all worldwide known genetically modified plant events, validated methods for event-specific identification were described. Especially in developing countries, adequate technologies for GMO assessment and authentication might not even be in place. The emergence of unauthorized GMOs (UGMOs) further complicate the issue. In the context of attack possibilities, what is critical is that they cannot easily be distinguished from authentic products (Holst-Jensen et al., 2012; Yang et al., 2013; Arulandhu et al., 2016).

Even in areas with rather strict regulations—as in Germany and France—it remains unclear how unauthorized GMOs can enter the food and feed chain. That is, how this commingling of seed happens, accidentally and unintentionally. Although the exact mechanisms are unknown, it may be possible that specific situations could be exploited for nefarious activities, such as differences and gaps in jurisdiction, limits of detection methods, or the deliberate release from field-trials.

An additional concern arises due to limited post-market analysis practices and regulations. While GMOs undergo critical risk assessment before approval, the presence of additional, deleted, or manipulated genes might not be obvious once such approval has been obtained. An attacker may be masquerading a manipulated (and hence, hazardous) as a certified GMO, or threaten to do so.

### 2.2. The Challenge of Detecting Counterfeit GMO Seeds

Traceability and labeling are critical factors to help authenticate GMOs. Currently, this is realized via unique identifiers. According to Bar-Yam et al. (2014), “A “unique identifier” refers to a simple numeric or alphanumeric code which serves to identify a GMO and to provide the means to retrieve specific information pertinent to that GMO. The codes may be used to access specific information on GMOs from a register, and to facilitate their identification, detection, and monitoring.”

Although such a code enables a genuine description of the product, this does not automatically validate the authenticity of the GMO itself. Already 15 years ago, it was realized that it would be crucial to have an identifier directly at the genomic level. This was realized via unique flanking sequences (Levine, 2004) and has become the basis for the most reliable GMO identification methods for decades.

### 2.3. How GMO Origination Can be Compromised

The very presence of unique identifiers of a GM plant (via, e.g., the event-specific characterization) might seem to validate the product under investigation. Unfortunately, this is based on a mistaken understanding regarding the functionality of such GMO signatures.

Traditionally, a signature string provides assurance of origination and content of a specific document, just as electronic signature and authentication methods guarantee integrity of an electronic sender and their products. However, with GMOs there is a real problem. Any self-authenticating signature element (even if it is enhanced by cryptographic methods, Mueller, 2014; Mueller et al., 2016) is an unreliable form of identification and is the very thing that may invite attacks. GMOs are not tamper-proof and an attacker can ingress changes (e.g., in form of genetic alterations leading to toxins or harmful products^3^) to existing—and certified—GMOs with the intent to invoke harm. An attacker can introduce changes without affecting the authenticating identifiers, allowing the adulterated product to pass as the real thing if only those identifiers are examined. For example, GMOs can be manipulated after risk-assessment and distributed as a counterfeit of the original certified product. If such manipulated GMOs masquerade as those which are approved, this will give a wrong assurance, both about the authenticity of the GMOs and the identity of the developing company or certification body.

### 2.4. Limits of Detection Methods

The problem is not only the manipulation of GMOs. What makes it worse is that practically these can rather easily be hidden inside the genome and that attacks may be done in a variety of settings (see below). Not only could plants be targeted via vectors directly in the field, it would similarly be possible to exploit gaps in the supply chain (Frazar et al., 2017) to replace authentic with manipulated seeds; or—on a small scale—agents could directly disperse counterfeit seeds.

Although numerous technologies have been developed to detect foreign DNA in GMO food and feed (see Bai et al., 2007; Chen et al., 2012; Holst-Jensen et al., 2012; Kamle and Ali, 2013; Arugula et al., 2014; Datukishvili et al., 2015), these procedures are still laborious, expensive, and time-consuming, not readily applicable for routine and rapid analysis in the variety of situations as described. Similarly, next generation sequencing (NGS) methods (see e.g., van Dijk et al., 2014; Goodwin et al., 2016) have their limits. For instance, they are complicated by unintended genomic changes which can occur within the transfer DNA (T-DNA) and its insertion (Schnell et al., 2015; Schouten et al., 2017) during the development of new GMOs (Park et al., 2017). Additionally, there are many other genetic changes that occur in plants both spontaneously and because of conventional breeding practices (see, e.g., Cao et al., 2011; Schouten et al., 2017). Consequently, due to the required sequencing depth and the huge quantity of data required, even NGS methods may not be readily applicable for the rapid type of analysis that would be required to detect clandestine manipulations of GMOs.

To alleviate regulatory concerns, CRISPR-based technology is gradually avoiding using transgene DNA. This is further complicating problems. An attacker could directly manipulate gene expression within a plant through dsRNA based post-translational gene-silencing methods (see the Discussion Section for technical and scientific challenges). Such modifications would be much more difficult to detect. Unless the dsRNA made by the GM plant is intended to act as a pesticide, the RNA itself is rarely formally considered in a risk assessment. As we are still lacking sufficient knowledge about the many novel RNA molecules (Heinemann et al., 2013; Arpaia et al., 2017) such intended manipulations would be extremely difficult to discover.

### 2.5. Hypothetical Scenario How These Vulnerabilities Can be Exploited

**Figure 2:**
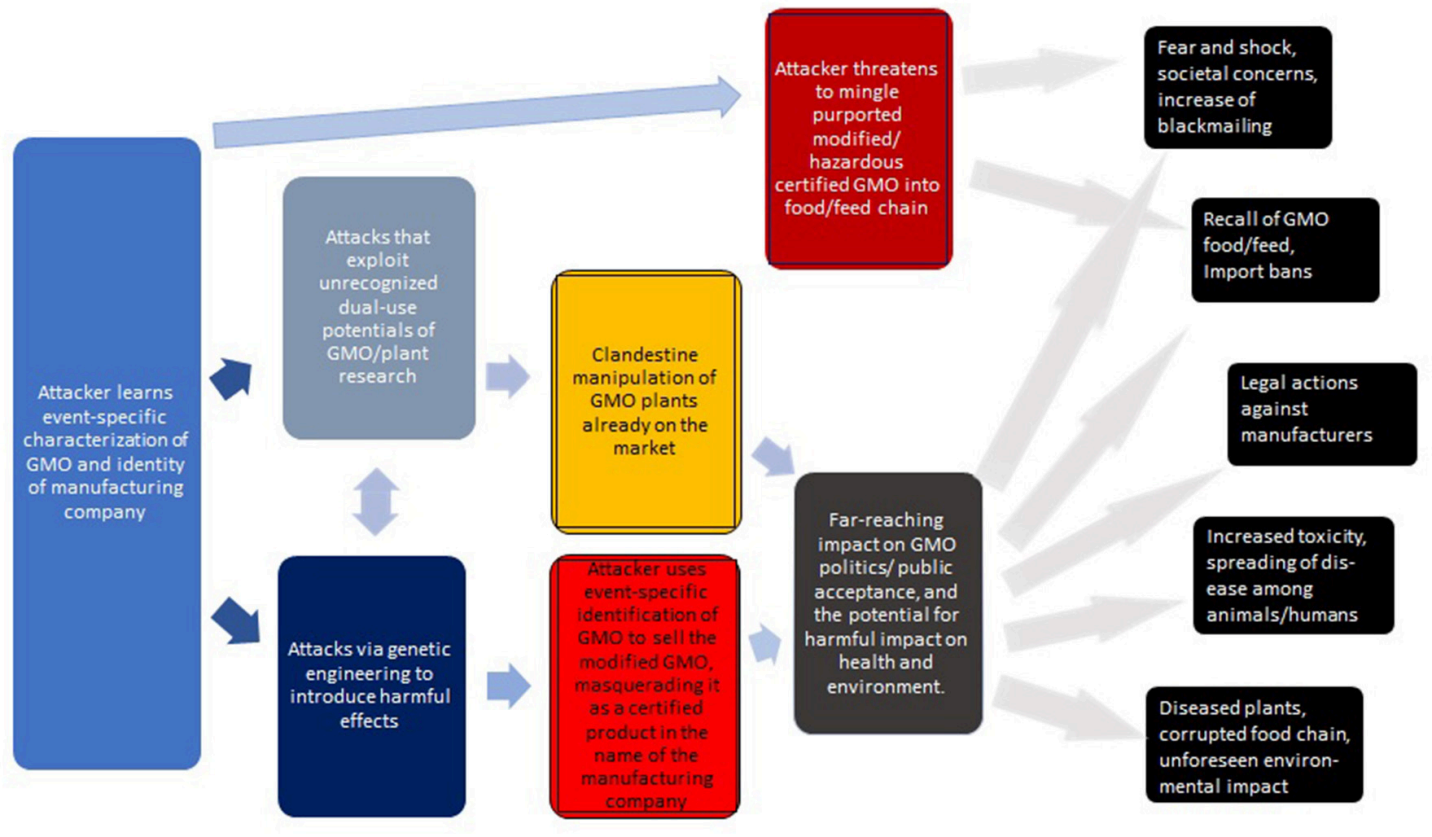
Generic overview of attacks via the clandestine exchange of GM plants with the intent to cause harm. The consequences on the right are roughly ordered from top to bottom in terms of feasibility and likelihood. The impact of some of these attacks may be profound.

It may be worthwhile to summarize the above (see Figure 2 for a generic overview). While each of the risks are significant, it is their combination that sets the stage for a new form of bioterrorism/biocrime involving the malignant counterfeiting of GMOs.

**Figure 3:**
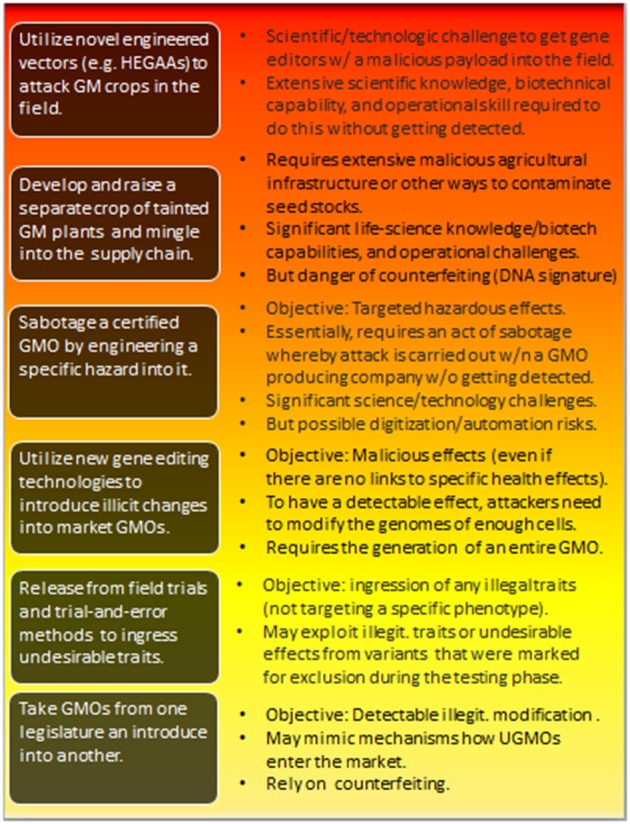
Types of potential attacks involving the hostile use of GMOs. The individual threats are roughly ordered from bottom to top in terms of increasing risk-potential—which correlates with the difficulties attackers are facing in effectively realizing those attacks. The impact is also hierarchical. Risks at the lower level are inherited at higher-level attacks. For the feasibility of the individual attacks and further discussions, see sections 4.2, 4.3, and 4.4. HEGAA, Horizontal environmental genetic alteration agents.

1. Agents seeking to perform the attack learn the event-specific characterization (typically, the flanking/border regions of the transgenes) of a specific GMO. (This information is publicly available).
2. The attackers wait until critical risk assessments and authentication processes—if existent in that jurisdiction—are completed (or performs the attacks in situations where these are not adequately supported). Alternatively, the attackers mimic some of the ways how UGMOs enter the market (Rostoks et al., 2019).
3. By utilizing novel technologies (e.g., CRISPR/Cas — see section 4 for scientific, technical, and operational challenges), attackers may exploit unrecognized insights from GMO and agricultural research to introduce various malignancies at the genomic or proteomic level (see Tables 3–5).
4. Depending on the intended scope and impact of these manipulations, the attackers can tailor how—and when—they can make their intrusions public (e.g., blackmailing, real threat, or mere hoax, see section 4.5 below and Figure 3).
5. It is critical to observe that the manipulated GMOs would still carry the authentic identifiers of the originals. Thus, with respect to these official identifiers—as could be verified by independent labs—the manipulated product would pass for the original.

Generalizing this, Figure 3 describes a hierarchy of attacks, including their key challenges (discussed in more detail below).

## 3. Specific Aims and Possibilities for Exploiting the Gaps

### 3.1. A General Overview of Potential Aims of Attacks

This section lists a variety of effects that attackers may be aiming at. These are summarized in Tables 1 and 2.

**Table 1:** Potential direct targets and attack aims.

**Attacks involving GM-plants**	**Manipulated/targeted effect and possible consequences**
An attacker could rely on traditional (e.g., *Agrobacterium mediated*) methods to transfer harmful viral or recombinant genetic material to target (selected) or off-target species, or “hijack” (genetically modify) viruses to perform gene editing of susceptible crops already planted.	(a) Designed spreading of toxins and harmful substances to targeted hosts (specific targets in the food chain). (b) Disruption of interactions between plants and their biotic environment (inflicting disease upon plants and indirect spreading of toxins).
Plants have innate players and mechanisms (toxins) to defend themselves against threats like pests and pathogens. The ability to produce toxins is need-based (some toxic plant pathways are inactive). An attacker could • upregulate a plant's natural toxin production, • downregulate their ability to defend themselves against their own pests/pathogens; (see also Table 4 below).	• Increased chemical toxicity from plants into feed and food products; tailor-made increase of existing (weak) mechanisms that make plants pathogenic to consumption (e.g., possible increased toxicity in additional plant parts and/or in various stages of the growing/harvesting/processing cycle). • Disease inflicted on plants by pests/pathogens and consequent contamination with mycotoxins; disruption of their microbiota; contamination of food and feed through toxic fungi or toxic soil bacteria. • Diseased plants cannot be utilized as source material for many industrial uses.
Specific promoters can not only upregulate the expression level but also lead to differential expression of transgenes in specific tissues (Arpaia et al., 2017).	• This may allow the (clandestine) introduction of toxins and harmful products, disguised as popular plant parts used for food and feed. • The (covertly introduced) or increased concentration of certain products in specific plant parts may disqualify them as edible crops or as a source material of specific industrial uses.
Targeted interference of cellular pathways, leading to an upregulation of immune reactions in those consuming the plant.	Disruption of immune response; induced hypersensitivity response to certain nutrients in animals and humans.

*The feasibility and impact of these types of manipulations are discussed in section 4.3, 4.2, and 4.4*.

**Table 2:** Potential off-target and indirect attacks.

**Attacks involving GM-plants**	**Manipulated/targeted effect and possible consequences**
The intended deletion or silencing of genes or mechanisms with plant protective properties.	The disruption of protective mechanisms (e.g., reduced levels of secondary metabolites, Arpaia et al., 2017) renders plants susceptible to toxic fungi or bacteria which could remain active through processes of food preparation.
Attacks in form of under-appreciated relationships and off-target effects. For instance, Bt crops express specific Cry proteins within the plant. The mode of action relies on interactions with specific midgut proteins of the targeted insect pest. An attacker may heighten or broaden the toxicity of Cry protein variants to interfere with new targets (see also Table 3).	The disruption of mechanisms involving off-target species (e.g., the human microbiome or viome), their relationships and synergistic effects.
An attacker may increase dominance of a new trait (e.g., through gene drives), or through misuse of infectious genetically modified viruses and other horizontal environmental genetic alteration agents, see Reeves et al. (2018).	Increased susceptibility to pests and pathogens; harmful effects on non-target-organisms; biodiversity disturbance, species displacement, and extinction; disturbance in soil micro-environment and species of ecological concern.

*See the Discussion Section for an analysis regarding their feasibility and impact*.

### 3.2. Dual-Use Potentials Fostered by GMO Research?

As known structures, including mobilization genes or origin sequences, are routinely screened in an attempt to ensure the safety of engineered products, an attacker may try to find unrecognized vulnerabilities often in form of new insights derived from GMO research.

It is possible that some of the most promising methods for supporting the utilization of GMOs may become a basis for attacks.

For instance, Table 3 considers some of the resistance management strategies that have been developed against the evolution of insect resistance to Bt toxins, and identifies that some of these may be actively misused.

**Table 3:** Bt toxins.

**Area of GMO research**	**Concern for their dual-use potentials**
To delay evolution of pest resistance to transgenic crops producing insecticidal proteins from *Bacillus thuringiensis* (Bt), the “pyramid” strategy uses plants that produce two or more toxins that kill the same pest (Wei et al., 2015).	**Key feature**: The rationale for such pyramids is that insects resistant to one toxin will be killed by the other toxin in the pyramid (Brévault et al., 2013). **Attack potential**: The pyramid could be turned upside down to achieve the opposite. For instance, Wei et al. (2015) show that two specific pyramided toxins work well in concert. However, one alone would automatically lead to cross-resistance. An attacker can “unstack” these carefully researched pyramided toxins and instead of the optimal combination replace toxins, say, with those exhibiting known antagonistic effects. **Consequences**: Instead of a delay in pest adaptation, such malignant transgenic crops might rapidly speed up evolution of resistance in several pests.
A new method to combat Bt toxin resistance was recently proposed by Badran et al. (2016). It is based on the following. • Bt toxins interact with protein receptors on the surface of insect midgut cells, leading to pore formation in the cell membrane and cell death (Adang et al., 2014). • It is possible to overcome Bt toxin resistance by evolving novel Bt toxins that bind with high affinity to new gut cell receptor proteins in insects (Badran et al., 2016). • The evolved Bt toxin variants very effectively alter toxin specificity, improve toxin potency, and bypass receptor-related resistance mechanisms.	**Key feature**: The approach demonstrated by Badran et al. (2016) enables targeting of a Bt-resistant pest through the evolution of high-affinity Bt toxin variants that bind a specific target insect protein. **Attack potential**: As anticipated by Badran et al. (2016), “In principle, this strategy should be applicable to target a variety of insect pests….” - or others? An attacker could instead target other sensitive proteins, including those of the plant itself, some of its microbes, insects and herbivores, or even humans. **Consequences**: There has been an ongoing controversy about the impact of Bt toxins on human health. Some have even claimed that Bt may have the capacity to puncture holes through the human digestive tract (Mesnage et al., 2013). With the new technology of *in vitro* evolution systems (which mediate the rapid laboratory evolution of diverse protein classes) an attacker might be able to achieve just that. As a result, they could be targeting gut proteins, not of the insect *Trichoplusia ni* (when it is consuming the plant), but of humans or animals that are consuming foods produced by the plant. The feasibility of targeting different species is supported by the following. While the evolved toxins obtained by Badran et al. (2016) only showed activity against closely-related insects species and maintained a similar insecticidal spectrum as the parental Bt toxin, an extension of their method allowed (Domínguez-Flores et al., 2017) to obtain novel variant with activities against distinct orders of insects.
A very effective way for delaying the evolution of pest resistance to transgenic crops is the “refuge-in-a-bag” approach, which consists of a random mixture of seeds of Bt and non-Bt plants of the same crop.	An obvious form of attack would consist in creating a different mix of seeds. For instance, as cross-resistance is expected to be stronger between toxins that are more similar (Carrière et al., 2016), an attacker could create a mix of Cry1, Cry2, and Cry3 toxins which all share a similar three-domain structure. While this will just defeat the purpose of the resistant management strategy, more dramatic situations might be possible via a similar mixed bag trick to introduce harmful features in clandestine.

*The evolution of insect resistance to Bt toxins is seen by many as one of the most serious threats to sustaining the gains offered by transgenic crops. This table considers some of the resistance management strategies that have been developed and investigates their possible dual-use potentials*.

Further, possible dual-use potentials of CRISPR/Cas technologies are listed in Table 4. A perpetrator may corrupt various plant-defense mechanisms to cause harm to the plant and its environment. Most importantly, some of these attacks may lead to toxic products which may get activated during harvesting or food processing (e.g., as corrupted canola oil), or which enter the food chain via indirect means (e.g., as mycotoxins following diseases actively inflicted on plants).

**Table 4:** Possible dual-use potentials of RNA-guided CRISPR-Cas9 systems to target plants.

**Key feature/potential dual-use component**	**Potentially adverse outcomes**
An attacker can direct Cas9 to cleave sequences involved in basic plant housekeeping mechanisms.	Weakening of plants, manifestation of disease, interference in plant interactions with other biota
*Fact*: In plants, miRNAs play an essential role in numerous developmental and physiological processes, such as fatty acid biosynthesis, growth and development, and responses to various stresses; many miRNAs are conserved across species (see Ding et al., 2018 and references therein).	Such a“miRNA stacking attack” may be targeted to deactivate the expression of critical genes in plants, which may be harmful to the plant itself and disrupt plant interactions with microbes, insects, and herbivores.
*Potential Target*: An attacker may silence specific miRNAs, or a combination of them, exploiting possible interactions or synergistic effects.	• In contrast to insect herbivory, the breakdown of certain plant secondary metabolites (glucosinolates) produced during the processing of oilseed meal have a harmful effect on animal thyroid function. The use of animal feed containing these glucosinolates has a negative effect on animal nutrition because of their goitrogenic properties (Borgen et al., 2010). • All forms of knowledge and techniques developed to minimize and compartmentalize toxicity could be turned upside down. An attacker may be able to do just the opposite. As a result, rather than being poisonous to only a few individuals who are eating specific plant parts whose toxicity gets triggered/enhanced through inadequate food processing techniques, the entire process could be corrupted by the means of modern gene-editors. A skilled upregulation of the toxins may lead to considerable health hazards for animals and humans.
*Potential Target*: Attackers may disrupt the natural—or even engineered—mechanisms plants use to defend themselves against pathogens. By utilizing gene editors like CRISPR/Cas, they may target the expression of key toxins (to disrupt their direct defense mechanism), or that of volatile substances that attract the natural enemies of their herbivores (to disrupt their indirect defense).
*Fact*: Apropos of direct defense, consider, e.g., oilseed rape and related crop plants and their defense system which has become known as the “mustard oil bomb.” • While many plants or plant products are poisonous, the toxins may not survive harvesting, processing, and cooking—and thereby not be of interest to bioterrorists. The uniqueness of the “mustard oil bomb” has been known for decades, as it has rather significant impact on human and animal health. • The toxic products may get released when canola seeds are pressed and lead to various health concerns among humans when the oil is consumed. • It has been possible to produce transgenic plants (Borgen et al., 2010) where the toxic compounds were (mostly) removed.
*Potential Attack*: An attacker may be able to do just the opposite and upregulate the production of these toxins.
Apropos of indirect defense and the expression of volatile substances that attract the natural enemies of plant herbivores. This is exemplified via a specific case-analysis. *Fact*: In maize, the contamination of plans with mycotoxins following fungal infection is a major problem. Among the mycotoxins, the aflatoxin B1 (produced by the fungal pathogen *Aspergillus flavus* “is the most carcinogenic compound found in nature”, Pechanova and Pechan, 2015). *Potential Target*: Ironically, new insights at the genomic/proteomic level might aid evil-doers. For instance, Pechanova and Pechan (2015) describe “tremendous differences between resistant and susceptible genotypes were observed in response to *A. flavus*” and quite a few other pests/pathogens. They were able to link the differences to specific induced and repressed proteins.	A number of detrimental effects of weakened expression of volatile substances—which would be the point of attack—are described by Pechanova and Pechan (2015) (for the case of maize), “grains contaminated with aflatoxins present immense agronomical problems leading to more than one billion of dollars lost annually…If not controlled, aflatoxins might be present in a wide range of maize-based foods and feeds, as well as in dairy products. They pose serious health hazards to both humans and animals, if digested via contaminated food and feed. In humans, aflatoxins have been directly linked to hepatocellular carcinoma, since they are metabolized in the liver…”.

*The consequences may be detrimental to the plant and pose serious health hazards to humans and animals*.

Finally, possible dual-use potentials of gene-drives^4^ are described in Table 5. The mechanisms of misuse described in this table extend those described above whereby plants are used as vectors to ingress illegal traits into the supply chain of GM crop seeds.

**Table 5:** Possible dual-use potentials of gene-drives.

**Key features/potential dual-use component**	**Potentially adverse outcomes**
Gene drives were originally invented for the alteration of sexually producing wild populations (Esvelt et al., 2014). Nonetheless, an attacker may construct gene-drives for plants that employ a mix of sexual and asexual reproduction. • The drive—especially with added malignant features—becomes a real issue in situations where such plants can form self-sustaining populations. • This may intentionally be pursued via targeted gene-transfer to produce transgenic × wild hybrids, or via strategic contamination of seeds of plants conducive to the development of GMO volunteers and feral populations. • As an example, feral oilseed rape is a known and widespread phenomenon (Pivard et al., 2008) and could become the platform to drive hazardous traits through plant populations.	The consequences of a gene-drive escaped into the wild are generally believed to be profound; or catastrophic, as gene-drives enable the spread of various traits, including malignant ones, such as disrupted defense mechanisms and resistance to various herbicides. Such feral plants could become virtually undestroyable weeds, and directly—through upregulated chemical toxicity—or indirectly—as host for toxic fungi or bacteria—become poisonous to humans or animals. At present, the impact of such hazardous drives on the environment and food chain is difficult to assess.

*The scenarios described in this table do not involve gene drives made within seeds used as part of the commercial seed supply, but involves other organisms outside the GM-supply chain that may interfere with crops as they grow. The impact of such attacks could be catastrophic*.

According to their original design (involving pest/pathogen populations in the wild), the intent of gene-drives is to drive a new trait through entire populations. The significance is that all the offspring of a gene drive organism that mates with, or is pollinated by, a natural organism will have the driven trait. Nonetheless, to affect a difference it is necessary that the drive can make its way through sufficiently many generations. Therefore, the impact of (manipulated) gene-drives in the context of cultivated crop plants seems minimal.

However, a new concern may arise when introducing a gene drive into a situation where plants are able to form self-sustaining populations, such as in non-GMO environments (see also section 4.7 below). This way, the attack vectors would not be commercial GM seeds, but in fact natural crop plants. Although the exact mechanism and impact of such attacks are open to debate, the consequences may be catastrophic.

### 3.3. Why Such Attacks May be Committed–on Motivations and Intent

Traditionally, security issues have been captured by cryptographic techniques which then expanded into various branches of cyber-security and information theory. While the biologic realm is also interested in (biologic) “information,” the latter involves many additional features than the one modeled by the cyber domain. Nonetheless, it seems beneficial to parallel possible motivations driving the threats within the cyber and the biologic domain. This is depicted in Table 6.

**Table 6:** On Motivations and Intent.

**Existing, known reasons for internet attacks**	**Possible reasons for bio-terrorist attacks involving GM plants (GMOs)**	**Technical and Operational Challenges**
Gain unauthorized access to information, in order to intimidate or coerce a government or its people in furtherance of political and social objectives.	Gain unauthorized access to power in form of blackmailing. A perpetrator may claim to have mingled manipulated seeds into the legitimate supply chain, and threatens to effect their targeted (or large-scale) release and distribution.	Low.
Impersonate another user or product for the purpose of: • originating fraudulent information, • modifying existing information, • fraudulently authorizing transactions or endorsing them.	Impersonate a developing company resp. a certified GMO for the purpose of: • originating hazardous GMOs, • modifying certified GMOs, • fraudulently selling certified GMOs.	High.
Impersonate another user or product for the purpose of: • creating fraudulent counterfeits, • attacking the reputation of that user when the illicit modification of the counterfeit is revealed.	Analogous.	High.
To cripple critical targets (based on political, social or religious objectives).	Diseases inflicted upon plants to harm the economy (or a competitor), to threaten the food supply, and pose health hazards on humans and animals (possibly with racist intentions).	High.
• Fraudulently restrict the license of others. • Causing others to violate a protocol by means of introducing incorrect information.	Introduce (a) illegal, or (b) harmful features into certified GMOs to evoke legal actions against biotechology companies and to bring certain producers or companies into discredit.	Medium (a), high (b).
Undermine confidence in a protocol or service by causing apparent failures in the system.	Undermine confidence in GMO production, sales, and politics.	Unpredictable.
Insider attacks (revenge, personal gain, personal or political motivations).	Analogous. This may include, (a) the release of inadequately performing GMOs during testing phases or trial-and-error experiments, or (b) the illicit release of those that have been manipulated in clandestine.	Low (a), high (b).
The challenge of breaking something that is believed to be secure; exploiting the naivety and ignorance of those susceptible to intrusion; being the first to demonstrate that attacks or security breaches are possible.	Analogous.	High.
Infliction of harm, (a) directly or, (b) with the aim of creating fear and shock).	Analogous.	Low (b), high (a).

*The cyber domain has been subject to possibly more attacks than most other areas. In order to comprehend possible motivations for attacks via GMOs, a comparison is made with attacks on the internet. In general, attacks involving biological materials are much more challenging to realize, especially in terms of large-scale effects (see sections 4.1, 4.3 for more details). Nonetheless, some parallels exist and need to be taken seriously*.

It should be stressed that the cryptographic view is radically different than the one prevalent in the biological/medical sciences. In the latter case, it is about saving and supporting life. It is difficult for those engaged in those disciplines to appreciate a kind of opposite driving force, such as the challenge of breaking a system. Anderson (2010) describes it this way, “Unless you're prepared to spend money …the mechanisms [developed to secure a system] will be defeated by people for whom it's an intellectual challenge…[to break these].”

## 4. Discussion

### 4.1. From CRISPR to the Production of GMOs

Concern has already been raised before regarding the—minimal—abilities required for modern synthetic biology technologies to be exploited in a malignant way. Frazar et al. (2017) write, “A non-state actor's ability to acquire or—depending on capabilities—generate products of concern using synthetic biology is improving because of rapidly maturing biotechnology techniques, technologies, and services. A person with basic knowledge of molecular biology and experience with gene editing techniques has access to a number of options from which to source desired material and design a fully functioning biological system.” Further, “In short, it is becoming increasingly easy for an adversary to not only acquire source material and design a sequence of concern, but also to synthesize that sequence and incorporate it into a living system.”

Furthermore, already a decade ago, Graham and Talent (2009) argued that “developing and dispersing a biological weapon would not be expensive—and it will only get cheaper and easier …The equipment required to produce large quantity …and then ”weaponize” the material—that is, to make it into a form that could be effectively dispersed—are of a dual-use nature and are readily available on the internet.” A few years later—after the emergence of CRISPR/Cas—Dunlap and Pauwels (2017) describe the situation as follows, “This ease has led the technology to already escape the lab, as companies currently sell kits targeted toward use in homes, and middle schools are using the technology in their science classes. These kits, for only $150, let you edit a bacterial gene using instructions made for those without expertise in little more than a weekend.”

The implications are sobering. Dunlap and Pauwels (2017) conclude that, “the emerging technologies …mean that access to the tools needed to create potential bioweapons are no longer maintained only with well-funded government or academic programs—a non-state group or rogue actor may be just as dangerous.” Similarly, DiEuliis and Giordano (2017) note that, “it is vital to acknowledge the existence of a robust “do-it-yourself” (DIY) community that already exists in biology, with both open community laboratories that are active around the country.”

Beyond doubt, the CRISPR-Cas system is a most effective gene-editing tool, and it is readily available. Although the system is targeting individual cells, it has been used to edit various organisms or cells from organisms. With respect to plants, editing efficiencies of the first CRISPR-Cas9 technologies for plant editing initially were relatively low (Mao et al., 2013) but have led to recent improvements (e.g., Ali et al., 2015; Globus and Qimron, 2018).

One of the key barriers to achieve noticeable effects lies in the delivery of the genome engineering reagents into plant cells. As plants are complex multicellular organisms, attackers need to find a way to manipulate sufficiently many cells to achieve the highest level of harmful effects (see Figure 3).

Although practically this can be realized via Agrobacterium-mediated transformation methods or alternatives (see e.g., Globus and Qimron, 2018), this means that in essence attackers have to go through the entire GMO production process. While this is a significant challenge, the potential for this cannot be ignored. Of special concern here are insider attacks (see Table 6) and underappreciated risk factors arising at the interface between biology and the cyber domain.

To illustrate this point, a rather benign incident was described by Peccoud et al. (2018). Specific plasmids were ordered by mail. After their arrival, a student immediately started measuring the expression of the genes encoded on the plasmid. After 6 months of unsuccessful attempts to reproduce the published data, sequencing of the plasmids revealed major discrepancies between the actual (physical) and published sequencing information.

The GMO production pipeline may harbor similar vulnerabilities. What an attacker can exploit is that a (digital) description of a product does not have to be the same as the product itself. As a result, skilled attackers could manipulate the underlying automation and digitization procedures to clandestinely tamper with authentic descriptions of the constructs encoding the CRISPR-Cas9 components.

The switching of authentic with fabricated genome engineering instructions is a serious threat (see also “Mapping the Cyberbiosecurity Enterprise” as a recent Frontiers Research Topic) and may have contributed to a critical feed contamination incident in the EU. In July 2014, Germany detected a viable genetically modified *Bacillus subtilis* strain in a vitamin B2 feed additive imported from China. The strain was identified as harboring a non-naturally occurring combination of DNA sequences. It is unauthorized in the European Union. Further analysis showed (Barbau-Piednoir et al., 2015; Paracchini et al., 2017) that the contaminating strain was not among those the manufacturer claimed to be using.

Correspondence between German diplomats, Chinese authorities, and the manufacturing company confirmed that there were critical genetic differences between the strains the company asserted to be using and those detected in Germany. Paracchini et al. (2017) were able to link these genetic modifications of unrecognized origin to specific plasmids described elsewhere in the literature. They concluded that “the production strain must have been contaminated or switched before or during production.”

Vulnerabilities arising at the interface of the digital and physical realm have hitherto received insufficient attention. They may indeed have played a role in the B2 feed contamination. Whether or not they can fully explain this incident, their potential for tampering with the GMO production pipeline cannot be ignored.

### 4.2. Operational Considerations

In order to induce any observable health effects among consumers (Figure 3), attackers misusing GMOs need to find a way to achieve a sufficient level of contamination. Assuming first that manipulations are introduced at the genomic level in such a way that they are able to induce substantial effects on the phenotype, attackers are then facing the challenge of introducing the manipulated GMOs into the food chain.

As previous incidents involving the import of unauthorized GMOs have shown, the potential of commingling manipulated seeds along similar routes must be taken seriously. Additionally, there is the risk about the mingling of seed from laboratories or field trials (see also section 4.3). Holst-Jensen et al. (2012) write, “The risk that people with access to un-authorized GMOs during development and field trials take seeds for own use or give away such seeds to others is not negligible. Furthermore, this risk is likely correlated with the perceived personal cost-benefit and negatively correlated with the educational level of the workers. The possibility that a GMO is escaping from field-trial releases into the environment and/or eventually ends up in the food supply chain without proper authorization therefore cannot be excluded.” Similarly, it is plausible that attackers remove variants with inadequate or illicit traits (see below).

### 4.3. Molecular Considerations and Challenges

As our mechanistic understanding of plant gene function and regulation is rather limited (see e.g., Rhee and Mutwil, 2014), trial-and-error methods are gaining increased importance in the engineering of modern crop improvement techniques. For example, to identify the optimal allele for a target trait, a guided trial-and-error approach based on genome editing was developed by Rodríguez-Leal et al. (2017).

The significance of this approach for attackers lies in the “error” outputs of the method. It is known that loss-of-function mutations disrupting genes may have extreme effects on the phenotype and also that the editing of regulatory elements can have unpredictable results (Rodríguez-Leal et al., 2017; Scheben and Edwards, 2018). Nonetheless, while it seems feasible that inside attackers could give away variants from among the pool of these “undesirable” variants, it would be considerably more challenging to undermine trials in order to obtain variants with targeted (intended) harmful phenotypic effects.

In spite of tremendous progress since the introduction of the CRISPR/Cas technologies just a few years ago, the simplest types of plant editing involves loss-of-function mutations into genes (Scheben et al., 2017). Mutations in *cis*-regulatory regions generally are expected to have quantitative effects (Wittkopp and Kalay, 2012), such as yield and fruit size, which may not be of critical interest to attackers intending to introduce toxins. On the other hand, qualitative changes in crops are more likely to be achieved by changing the product of a gene (Scheben and Edwards, 2018). Moreover, additional influences of gene regulation have been identified, such as transcription factors (Jin et al., 2013) and chromatin changes. These may even be more effective at altering the function of regulatory elements than the small indels produced by Cas9 that often have no effect (Canver et al., 2017).

Thus, as predicting the phenotypic consequences of a specific mutation *in silico* is rarely possible, the challenges for attackers trying to achieve specific harmful effects are significant. Yet, it needs to be stressed that different types and levels of (arbitrary) modifications may be useful to attackers nonetheless (see Figure 3).

### 4.4. Risk-Assessment and Impact

To the best of my knowledge, the types of risks introduced in this paper have not been described before. The lack of familiarity with new technologies presents a challenge to risk analysts who wish to precisely identify the hazards and the likelihood and magnitude of these outcomes. “The Regulation of Synthetic Biology—a Guide to United States and European Union Regulations, Rules and Guidelines” (Bar-Yam et al., 2014) articulates the predicament of synthetic biology and new technologies, “ The problem of developing methods for appraising risks and benefits associated with increasing novelty has yet to be addressed.”

While new technologies have been leading to important insights how to advance plant breeding and agricultural issues, these may establish unrecognized risk potentials. Tables 3–5 describe how some of this knowledge may be turned upside down and become a basis for attack. For instance, Borgen et al. (2010) provide detailed genomic insights about the expression of toxins. However, this does not mean that perpetrators merely have to modify the genes involved, and that they will automatically achieve—in a precisely predictable way—a level of toxicity that is sufficiently detrimental (either to the plant itself, to those consuming it, or in terms of economic impact). Nonetheless, some critical factors cannot be overlooked.

Attackers may pursue various levels of attacks (see Figure 3), ranging from mere hoax (not included in Figure 3) to the sabotaging a biotechnology company and selling corrupted GMOs intended to harm the agar and food sector. In the latter case, attackers would literally have to design their own GMOs and bring them into large-scale distribution. This is not a simple task. While there has been enormous progress since the first generation of GMOs, modern technologies nonetheless require significant amount of lab work and field trials.

It should be noted, though, that attackers would pursue opposite goals than those pursued during legitimate GMO production. The latter is concerned with biocompatibility issues and regards unintended risks to health and environment. These practices are aimed at ensuring that the new traits get introduced without leading to unexpected effects or harming non-target organisms. Moreover, a great challenge in producing CRISPR-based GMOs lies in poor CRISPR-Cas specificity leading to frequent off-target editing. This must be avoided if regulatory approval is required and considerable effort has been devoted to alleviate this problem. Such an orientation is singly minded toward the achievement of very precise goals—targeted and critical phenotype expression levels of the intended modification. Everything else other than the specific goals would be unacceptable.

Yet, attackers would be dealing with the opposite scenario. Possibly, they do not even want a (targeted) effect (see Figure 3). They don't need to pass critical regulatory protocols to prove specific causalities and relationships. Even if they were aiming at the targeted expression of key genes, say, any possible side effects could work to their advantage.

Even if the clandestinely introduced alterations did not lead to the anticipated linear causal relationships (i.e., the intended harm), they could have unexpected effects that are detrimental nonetheless. These negative effects may involve the plant itself, but could be on a more general level, from cellular to political and economic. A critical factor here is public opinion. According to Parrott (2018), non-GMO is the fastest growing sector of the US food market at present. The very claim of a clandestine manipulation (possibly even of a non-GMO) cannot be ignored as it may have further impact on public acceptance of GMO politics and regulations. In legislatures with strict anti-GMO politics, threats such as blackmailing could have a drastic impact.

The very announcement that one, or possibly many, manipulations have happened, may lead to considerable unease and angst. Attackers could further stir the fear of the public by claiming that some of the introduced modifications are intentionally disguised within the genome, or that some of the biochemical pathways to introduce toxic effects are clinically significant but difficult to diagnose. Take the example of chemical toxicity described in Table 4. The claimed effects might include medical symptoms such as leaky gut, thyroid damage (Pechanova and Pechan, 2015), and autoimmune disease. Nonetheless, the very presence of these symptoms does not mean that the purported hazardous GMO was the causative agent.

Now add to this the problem of analytically identifying the purported hazardous gene edit. New breeds of GM crops have led to substantial debates regarding their biosafety, commercial use, and regulation. The very fact that attacks through and on plants (possibly extending to non-GMOs, see section 4.7) are difficult to detect would yet further increase public unease and increase doubt in these new technologies and the companies producing them.

### 4.5. Disclosure of the Attacks

As there is limited comprehension about many biocrime risk potentials, this may be exploited by those seeking to do harm. Dunlap and Pauwels (2017) raise one important issue, “Using current capabilities and available resources, it may be possible to detect [various forms of biocrime]…but would likely take substantial time (weeks)…In the event of an intentional biological attack, this is far too long of a period to detect and assess.” Similarly, Murch (2015) stresses the importance of getting caught, as “Getting caught and being held accountable, or the credible threat thereof, “raises the bar” for those who engage illicit activities related to biowarfare, bioterrorism, or bioproliferation.”

Depending on their goals (Figure 3), attackers might prefer to remain undetected. For instance, when trying to evoke public unease or undermine the confidence in bioengineering and GMOs, the emergence of contaminated products will likely increase this effect, even more so the harder it is to determine the exact origin of the manipulation. This point was clearly demonstrated by the Amerithrax attacks which resulted in years of investigations and billions of dollars spent toward biodefense efforts. All that attackers would need to do to stir public angst, is to make sure that unauthorized GMOs (whether truly hazardous or not) will be detected (perhaps during routine screening).

The challenge of rapid and efficient identification of manipulated GMOs could additionally be exploited via blackmailing attacks. In this case, perpetrators would just need to provide sufficient evidence regarding the plausibility of the attack. In order to do that, attackers would only need to make sure that some modifications get detected, e.g., via genetic or chemical analysis. Such threats cannot be taken lightly. Given that both the authentic and the manipulated GMOs would carry the same DNA signature elements, the extent of the intrusion—from targeted and isolated manipulations to sabotaging the manufacturing company—would not immediately be clear.

Concerns about GMO have had a significant impact on public opinion and disputes over unauthorized GMOs are known to have considerable effect on economy and trade (Holst-Jensen et al., 2012). Now add to this the recent series of trials and law-suits (e.g., Reuters, 2019) against some of the biomanufacturing companies. Thus, blackmailing attackers could capitalize on the very fact that the exact extent of their purported threats would be difficult to assess.

### 4.6. The Scope of This Study

While modern technologies may pave the way for various forms of biocrime involving plants, the potential of counterfeiting makes GMOs even more vulnerable. The critical point is how these attacks could get detected. If manipulations could easily be identified, then they would be less “attractive.” Here is where the predicament of counterfeiting comes in. For decades, the problem of GMO authentication seemed to be solved, and methods utilizing the unique flanking sequences were believed to be most reliable. Unfortunately, due to the advancement of modern gene editors and technologies, this is no longer case.

A much easier way to introduce harm than discussed herein would consist in genetic manipulations to develop more virulent or resistant pests/pathogens in order to harm plants or their microbiota. When introduced in form of manipulated gene-drives, this would have a large-scale detrimental impact on plants as well as non-target organisms and have a cascading effect on the entire food-web. At present, the potential for manipulating gene-drives is difficult to assess.

The reason that GMOs are considered in this study lies in the identification issues. By clandestinely exchanging GMOs with adulterated versions, and by actually utilizing and verifying the authentic identifiers, an evil-doer appears in sheep's clothes. Although protocols utilizing flanking sequences are particularly vulnerable, the same is true for other methods. As long as there is no easy and rapid way to authenticate GMOs, this may enhance the scaled-up distribution of hazardous counterfeits.

As this is the first study of this kind, the focus was mainly on GM plants. The full extent of biocrime vulnerabilities involving biological mediums, including animals and viruses, needs to be further analyzed (see, e.g., Reeves et al., 2018).

### 4.7. Open Questions

Skilled attackers might be able to exploit numerous insights about functional relationships, synergistic effects and emergent properties, plasticity of gene expression and memory effects, or under-appreciated contributors such as the active role of proteins or metabolism, the human/plant microbiota, and epigenetic regulation. The list seems endless. Nonetheless, without a mechanism to scale-up the intrusion, many attacks might only have a rather limited—that is, local—impact. The discovery of gene drives (Esvelt et al., 2014) might change this all—at least in circumstances in which the seeds from a crop into which a gene drive has been inserted can be used to grow a subsequent generation of crops.

Although biosecurity issues of gene drives have been the subject of several studies (e.g., Oye et al., 2014; Lunshof and Birnbaum, 2017), the risks related to intentional attacks via the release of—possibly several concomitant—drive-containing plants have not been adequately assessed. Of special concern here is the deliberate manipulation of drives, to introduce harmful features into non-GMO plants in areas where farmers rely on their own seeds rather than on commercial seed production. Another question relating gene-drives is which, and under what circumstances, locally popular plants (such as maize in Austria, see Pascher, 2016) are able to quickly form self-sustaining populations outside cultivation. The occurrence of volunteer plants in subsequent crops and feral plants in non-agricultural areas is a well-known risk factor involving GM plants but has not been adequately assessed relative to their potential for active misuse. Engineered with various resistant traits—purposefully targeted and designed to exploit existing climate and geographic stressors—the question now is if they could take over both native and GMO-maize, say, and corrupt any edible maize populations, and possibly even be created to cross over to related species, thereby wiping out the entire crop production in a certain geographic area? A similar question exists regarding the engineered cross-over of chemical toxicity, into plants (or parts) that previously have been edible.

Related questions arise relative to horizontal gene transfer (HGT). While the frequency of (unintended) HGT has been under debate, certain transgene features are known to increase introgression into wild counterparts (Tsatsakis et al., 2017 and references therein). It is not clear to what extent these may be targeted by skilled attackers—who might try to corrupt nearby non-GMOs. A more sinister type of attack involves the direct genetic manipulation of non-GMOs (or those marketed as such). Since nobody would be looking for them, it is not clear how such attacks could be most effectively detected.

Apropos of GMO authentication. One of the most critical questions would be to develop a rapid and effective GMO method that, (1) provide a signature type function which, (2) cannot be copied or transferred, and which (3) detects arbitrary manipulations that might be hidden inside the GMO. Given that GMOs are not a “closed box” it seems that such signatures would have to rely on something different than just the genomic sequence information. Extending the signature paradigm to the various omics settings seems like a daunting task, especially in light of the plasticity and flexibility that is governing natural processes.

## 5. Conclusion

This article suggests that criminals may exploit modern gene editing technologies via new forms of attacks that involve the clandestine manipulation of market GMOs (or the malicious insertion of genetic modifications into ostensibly unmodified plants). The identification and analysis of the harmful genetic manipulation of plants as an attack vector shows that these concerns need to be taken seriously, raising the prospect not only of direct harm, but of the more likely effects in generating public concern, reputational harm of agricultural biotechnology companies, law-suits, and increased import bans of certain plants or their derived products.

If an agent can manipulate legitimate GMOs, the emergence of undesirable traits amongst GM plants and their derived products might not immediately point to an attack. Disguised as a certified product, researchers might look for other reasons to explain such effects, trying to find reasons via unrecognized natural phenomena or biochemical pathways. The investigations might point away from the reality of an attack, let alone point to the true identity of the attacker. It might take a while until the DNA of the harmed plants would become the target of investigation, and when, say, full-genome sequencing would point to the presence of the foreign gene(s) (if existent) responsible for the observed, new, harmful traits. However, attacks based on more recent technologies that influence the proteome or metabolome would be much harder to detect. Similarly, the effectiveness of authentication and surveillance protocols may be overridden by skilled misuse of newer technologies such as gene-drives, phage assisted methods, or *in vitro* evolution techniques.

While some of the scenarios described may be a bit far-fetched, this was done with the intent to anticipate a large range of approaches that potential attackers might be pursuing. This paper should be seen as an invitation to start a dialog and to raise awareness. It is the type of study that never realized would enjoy it's greatest success.

## Author Contributions

The author confirms being the sole contributor of this work and has approved it for publication.

### Conflict of Interest Statement

The author declares that the research was conducted in the absence of any commercial or financial relationships that could be construed as a potential conflict of interest.

## Footnotes

^1^The Centers for Disease Control and Prevention (CDC) is the leading national public health institute of the United States, https://www.cdc.gov/.

^2^for simplicity, herein referred to as GMOs.

^3^Throughout, “harmful GMOs” are meant as a result of attacks that deliberately introduce harmful features.

^4^I am indebted to the reviewer who suggested to include gene-drives into the present analysis.
